# Therapeutic potential of fennel essential oil and manganese in modulating steroidal hormonal imbalance and ovarian alterations in rats with polycystic ovarian syndrome: An experimental study

**DOI:** 10.18502/ijrm.v23i1.18201

**Published:** 2025-03-21

**Authors:** Ensiyeh Mohebbi Kian, Maryam Barancheshmeh, Hossein Najafzadehvarzi, Seyedeh Masoumeh Ghoreishi, Naser Shokrzadeh

**Affiliations:** ^1^Department of Pharmacy, Ayatollah Amoli Branch, Islamic Azad University, Amol, Iran.; ^2^Pharmacology Department, Faculty of Medicine, Babol University of Medical Sciences, Babol, Iran.; ^3^Cellular and Molecular Biology Research Center, Health Research Institute, Babol University of Medical Sciences, Babol, Iran.; ^4^Reproductive Health Research Center, Clinical Research Institute, Urmia University of Medical Sciences, Urmia, Iran.

**Keywords:** PCOS, Rat, FEO, Mn, Oxidative stress, Estrogen, Progesterone.

## Abstract

**Background:**

Polycystic ovarian syndrome (PCOS) is a prevalent hormonal disorder among women of reproductive age, resulting in female infertility. Researchers are exploring safe and affordable treatments for this disorder.

**Objective:**

This study explored the therapeutic effects of fennel essential oil (FEO) and manganese (Mn) on hormonal and histological markers in rats with estradiol valerate-induced PCOS.

**Materials and Methods:**

In this experimental study, 35 adult female Wistar rats (9 wk old, 200–250 gr) were divided into 7 groups. For 14 days, groups 1–4 received normal saline intraperitoneally, sesame oil intramuscularly, FEO intraperitoneally, and Mn orally, respectively. PCOS was induced in remaining groups through a single intramuscular injection of estradiol valerate. 60 days after induction, the 6^th^ and 7^th^ groups were treated individually with intraperitoneal FEO and oral Mn for 14 days. Blood samples were analyzed for estrogen, progesterone, and malondialdehyde (MDA) markers. The ovarian tissues were histologically examined to assess cyst formation and structural changes.

**Results:**

FEO significantly increased estrogen and progesterone levels in PCOS rats compared to the PCOS group (p ≤ 0.05). Mn also elevated progesterone levels, but the change was not statistically significant (p > 0.05). No significant differences in MDA levels were observed between the PCOS and PCOS+FEO groups. Although MDA levels decreased in the PCOS+Mn group, the reduction was not statistically significant (p > 0.05). Both FEO and Mn treatments significantly reduced ovarian cyst numbers compared to untreated PCOS rats (p ≤ 0.05).

**Conclusion:**

FEO and Mn demonstrated potential in restoring hormonal balance and improving ovarian histology, offering promise as low-cost treatments for PCOS.

## 1. Introduction

Polycystic ovary syndrome (PCOS) is a common hormonal disorder affecting 8–21% of reproductive-age women (1), with symptoms like hair loss, hirsutism, acne, menstrual irregularities, and infertility (2). Although the exact etiology of PCOS is unknown, several genetic and environmental factors may play a role, including hormonal imbalances, ovarian steroid synthesis problems, oxidative stress, nutritional deficiencies, obesity, and insulin resistance. Among these, oxidative stress and hormonal imbalances, particularly in estrogen and progesterone, are increasingly recognized as key factors (3). This emphasizes the urgent need to develop effective, safe, and affordable treatment options for PCOS.

Hormonal imbalances, such as the elevated levels of luteinizing hormone (LH), accompanied by decreased levels of follicle-stimulating hormone both disrupt the androgen production and estradiol synthesis in the ovaries (4) causing follicular atresia, anovulation and low progesterone levels as well (5). In PCOS women, the conversion of androgens to estrogen in adipose tissue worsens hormonal imbalances (6). Additionally, these changes can be aggravated by oxidative stress, which is linked to excessive free radicals and inadequate antioxidant capacity (7). These contributing factors, together, promote the development of PCOS, although the precise mechanisms are still unclear.

Existing treatments for PCOS, including lifestyle interventions, pharmaceuticals, and surgery, are often associated with side effects and high costs. Therefore, alternative approaches utilizing natural products like medicinal plants and micronutrients are gaining attention due to their safety profiles and cost-effectiveness in therapeutic applications (8–10). *Foeniculum vulgare* (fennel), with its phytoestrogenic and antioxidant properties, and manganese (Mn), a micronutrient crucial for antioxidant enzyme activity and hormone regulation, are promising candidates (11). Mn is a vital micronutrient implicated in oxidative stress pathways (12). It participates in steroid hormone synthesis and therefore plays a role in reproductive function (13). With Mn supplementation, serum LH, follicle-stimulating hormone, and estradiol levels markedly increased demonstrating that Mn is important for hormonal regulation (14). However, studies on serum Mn levels in PCOS patients have produced conflicting results, with some showing deficiencies (12–14) and others reporting elevated levels (15). Upon exploring Mn, fennel drew particular attention due to its phytoestrogenic, anti-inflammatory, and antioxidant properties (16). The phytoestrogenic compounds in fennel, especially anethole, are known to alter hormonal balance by raising prolactin secretion and estrogen and progesterone levels (17). By integrating fennel into therapeutic protocols, it may be possible to develop more effective approaches to managing PCOS and related hormonal disorders along with antioxidant effect (18, 19). Approaching oxidative stress and hormonal imbalances with fennel and Mn represents a very intriguing therapeutic pathway in the treatment of PCOS, which clearly presents prospects for further investigation.

There is an urgent need to explore innovative alternative treatment approaches, due to the effect of PCOS on female fertility and overall health. This study attempts to fill the critical gap regarding alternate treatments by determining the effects of fennel essential oil (FEO) and Mn. While FEO and Mn have been studied independently in PCOS, no study compares their therapeutic effect directly. In contrast to former researches, our investigation offers an innovative comparative study of FEO and Mn that evaluates which one of them is more efficient in restoring hormonal equilibrium, lowering oxidative stress (as indicated by the malondialdehyde [MDA] levels), and ameliorating ovarian damage in PCOS. As the most obvious gap in the research, this helps understanding the mechanisms of action, and the scope for the management of PCOS. This work extends the current understanding by elucidating the therapeutic potential of these agents and also paves the way for developing safer, effective, and evidence-based treatments for PCOS.

## 2. Materials and Methods

### Animals

In this experimental study, which was conducted at Babol University of Medical Sciences, Babol, Iran between September 2021 and January 2022, 35 adult Wistar female rats (about 9 wk old, weighing 200–250 gr) were enrolled. The rats were kept in standard polypropylene rat cages with a 12-hr light/dark cycle, controlled for temperature between 20–24 C. They were loaded with pellet food and clean tap water on a limitless basis. The number of animals in every experimental group was limited to the least possible to alleviate discomfort and pain.

### Chemicals

Estradiol valerate (EV) was purchased from Aburaihan (Tehran, Iran). Sesame oil and FEO were obtained from Barij Essence (Kashan, Iran). Mn sulfate, ketamine, and xylazine were sourced from Merck (Darmstadt, Germany). MDA and steroid kits were provided by Kiazist Company, Iran.

### Experimental design

To conduct a comparative evaluation, 35 intact female rats were randomly assigned to 7 groups (n = 5/each).

At 60 days of age, the animals were randomly allocated to the following groups and received below-mentioned regimens for 14 consecutive days:

1. The control group received 1 mL of normal saline intraperitoneally.

2. The sesame oil group received 0.1 mL of sesame oil (vehicle for EV) intramuscularly.

3. The FEO group received 200 mg/kg of FEO intraperitoneally.

4. The Mn group received 10 mg/kg of Mn via oral gavage.

**Figure 1:**
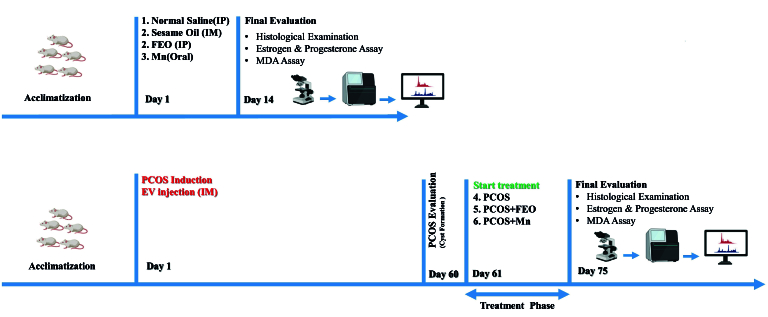
Overview of the experimental timeline and methodologies. The upper part of the figure outlines the experimental setup for 4 groups, which were given respectively normal saline (control), sesame oil (vehicle), FEO, and Mn daily for 14 consecutive days. The lower part shows the protocol for inducing PCOS through a single intramuscular injection of EV (4.0 mg/kg) on day 1, followed by a 60-day period to allow for cyst formation. On day 61, the treatment groups received daily doses of 200 mg/kg FEO (intraperitoneally) and 10 mg/kg Mn (orally) for 14 days. At the end of this treatment phase, ovarian tissues were collected for microscopic analysis, estrogen, progesterone, and MDA assay. IP: Intraperitoneally, IM: Intramuscularly, FEO: Fennel essential oil, Mn: Manganese, MDA: Malondialdehyde, PCOS: Polycystic ovarian syndrome, EV: Estradiol valerate.

In the remaining groups, a PCOS condition was induced by administering EV via a single intramuscular injection (4.0 mg/kg diluted in 0.2 ml sesame oil) during the rat's estrous cycle. EV is a synthetic compound that can effectively mimic the morphology of PCO. The animals were randomly assigned to one of the following 3 experimental groups (as indicated in Figure 1):

5. PCOS group received no further treatment.

6. PCOS+FEO group treated with 200 mg/kg of FEO intraperitoneally for 14 consecutive days (17).

7. PCOS+Mn group treated with 10 mg/kg of Mn via oral gavage for 14 consecutive days. Mn was ground into a fine powder using a homogenizer and diluted in water.

### Sample preparation

One day after the final injections, adhering to the ethical standards for working with animals, the rats were euthanized using ketamine (80 mg/kg) and xylazine (5 mg/kg) administered intraperitoneally. Blood was then collected via cardiac puncture. An additional dose of ketamine was given to ensure a painless and rapid euthanasia process. The rat's serum was subsequently separated and centrifuged, and its values for various parameters were measured using specific kits and an enzyme-linked immunosorbent assay reader.

#### MDA assay

To determine lipid peroxidation resulting from oxidative stress, an MDA assay was performed. MDA, a byproduct of lipid peroxidation, reacts with thiobarbituric acid at high temperatures to form a pink chromogenic compound (20). This compound is measured calorimetrically at wavelengths of 530–540 nm using an enzyme-linked immunosorbent assay reader. The MDA values in the samples were determined using a standard curve.

#### Estrogen and progesterone assay

The amounts of estrogen and progesterone in the serum of the rats were determined using an animal kit provided by Pars Azmoon Co., Iran, in a medical laboratory.

### Outcomes

The primary outcome was to compare the effects of FEO and Mn on the levels of steroidal hormones and MDA in experimental and control groups. The secondary outcome included comparing the number of cysts in the experimental groups.

### Histological analysis

After the rats were sacrificed, their ovaries were isolated, washed with phosphate-buffered saline, and fixed in 10% formaldehyde. For tissue staining, the slides were stained with hematoxylin and eosin and examined under a light microscope (Olympus, Japan). A quantitative assessment was conducted by counting the number of cysts in each section of the ovary 3 times.

### Ethical Considerations

All experimental procedures and protocols were approved by the Animal Ethics Committee of Islamic Azad University, Ayatollah Amoli Branch, Amol, Iran (Code: IR.IAU.AMOL.REC.1400.019) with the Guide for the Care and Use of Laboratory Animals published by the United States National Institutes of Health (NIH Publication, 8^th^ Ed, 2011).

### Statistical Analysis 

The analysis of data with differentiation of experimental groups was done by Statistical Package for the Social Sciences, version 24 (IBM, Armonk, NY, USA). For the evaluation of statistical significance, a one-way ANOVA (analysis of variance) with post hoc Tukey's test was used. The participants were provided with Mean ± SD and it was only deemed significant if p ≤ 0.05.

## 3. Results

In examining the condition of the rats during the research, no fatalities were observed, and no visible disorders or diseases were detected.

### Estrogen level in the experimental groups

**Figure 2:**
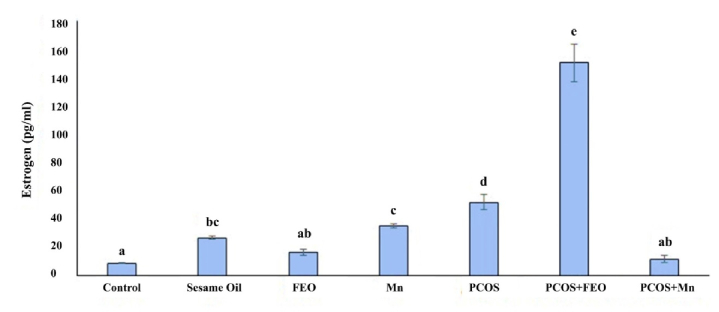
Estrogen level in the serum of rats in different groups. The same letters between the 2 groups show that the 2 groups are not statistically different. P ≤ 0.05 is considered statistically significant. FEO: Fennel essential oil, Mn: Manganese, PCOS: Polycystic ovarian syndrome.

Regarding the Mean ± SD of estrogen levels in the serum of rats in different groups, the highest amount was reported in the PCOS+FEO group, at 150.5 ± 23.5 pg/ml, while the lowest amount was observed in the control group (which received normal saline) at 8.56 ± 0.55 pg/ml. The differences between the control group and the PCOS+FEO group, as well as between the PCOS+FEO and PCOS+Mn groups, were statistically significant (p ≤ 0.001). Additionally, the Mean ± SD of serum estrogen levels in the group that received sesame oil was statistically significant compared to the control group (p = 0.04), the PCOS group, and the PCOS+FEO group (p ≤ 0.001) (Figure 2).

### Progesterone amounts in the experimental groups

**Figure 3:**
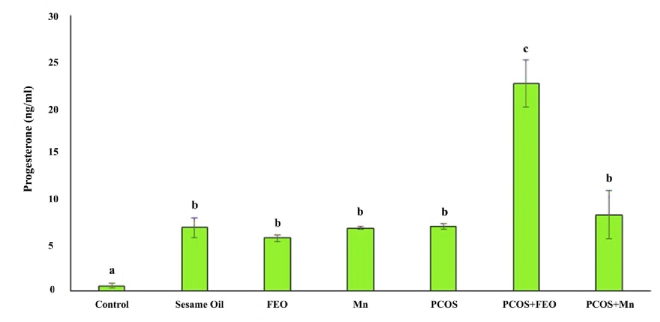
Progesterone level in the serum of rats in different groups. The same letters between the 2 groups show that the 2 groups are not statistically different. P ≤ 0.05 is considered statistically significant. FEO: Fennel essential oil, Mn: Manganese, PCOS: Polycystic ovarian syndrome.

Figure 2 shows the Mean ± SD of progesterone levels in the serum of rats in different experimental groups. The highest level was observed in the PCOS+FEO group at 22.6 ± 4.4 ng/ml, while the lowest level was 0.57 ± 0.48 ng/ml in the control group. The progesterone level in the control group was significantly lower than in all other groups (p = 0.02). The PCOS+FEO group had a significantly higher progesterone level compared to all other groups (p ≤ 0.001). However, no statistically significant difference was observed in progesterone levels between the group that received sesame oil, FEO, Mn, the PCOS group, and the PCOS+Mn group (Figure 3).

### MDA amounts in the experimental groups

**Figure 4:**
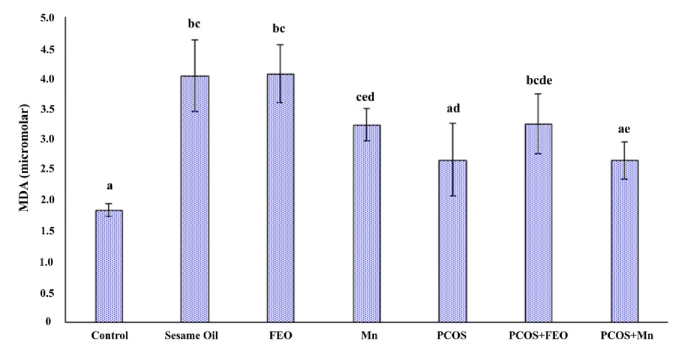
Amount of MDA in the serum of rats in different groups. The same letters between the 2 groups show that the 2 groups are not statistically different. P ≤ 0.05 is considered statistically significant. FEO: Fennel essential oil, Mn: Manganese, MDA: Malondialdehyde, PCOS: Polycystic ovarian syndrome.

Regarding the Mean ± SD of MDA levels in the serum of rats in different groups, the highest value was observed in the group that received FEO (4.08 ± 0.82 µM), while the lowest value was seen in the control group (1.84 ± 0.17 µM). The differences between the control, sesame oil, and FEO groups were statistically significant (p ≤ 0.01). Additionally, the difference between the control group and the PCOS+FEO group was statistically significant (p = 0.04). However, the difference in MDA levels between the control group and the PCOS+Mn group were not significant (Figure 4).

### Histological assessment

For further confirmation, histological studies were performed. In the histological examination of ovarian tissue in the microscopic section with hematoxylin and eosin staining, corpus luteum, ovarian cysts, and various types of follicles, particularly secondary and Graafian follicles, were observed.

**Figure 5:**
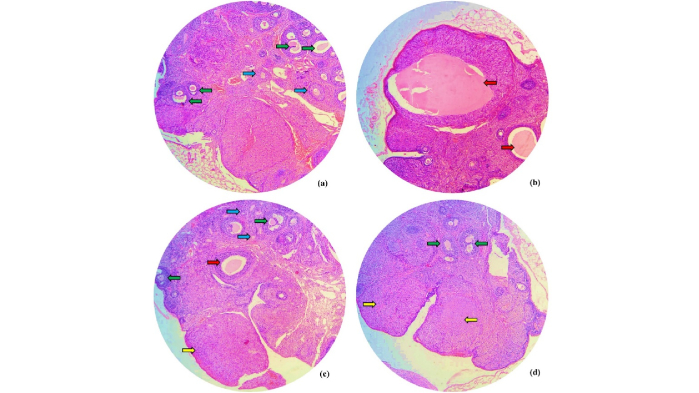
Histological results of the ovary in a) Control, b) PCOS, c) PCOS+FEO, and d) PCOS+Mn groups. Yellow arrow: Corpus luteum, Red arrow: Cyst, Blue arrow: Primordial follicle, and Green arrow: Secondary (antral) follicle.

As seen in figure 5, PCOS was successfully induced in the group that received EV (Figure 5b). The picture clearly shows that the PCOS group had more and larger ovarian cysts compared to the control group (Figure 5a). The histological study results indicated that the number and size of ovarian cysts were reduced in both the PCOS group treated with FEO (Figure 5c) and Mn (Figure 5d) compared to the PCOS group.

Ovarian cysts in different study groups are represented in table I. The results show that the average number of ovarian cysts for the group that was treated exclusively with FEO was significantly higher than those of all other groups (p ≤ 0.001). In the PCOS group, where ovarian cysts were created by EV injection, the mean number of cyst was significantly lower than the FEO group (p ≤ 0.001). On the other hand, in the PCOS+FEO group, the mean number of cysts was significantly lower than that of the PCOS group (p ≤ 0.001). Also, the group received only Mn had significantly higher number of ovarian cysts than the control group (p ≤ 0.01). In contrast, the PCOS+Mn group had statistically significantly lower cyst number than the PCOS group (p ≤ 0.001).

**Table 1:** Number of ovarian cysts in different groups

**Variable**	**Group**						
	**Control**	**Sesame oil**	**FEO**	**Mn**	**PCOS**	**PCOS+FEO**	**PCOS+Mn**
**Number of ovarian cyst **	0.00 ± 0.00	0.00 ± 0.00	7.33 ± 1.53^a^	2.00 ± 0.00^b^	4.33 ± 0.33^c^	2.00 ± 0.00^b^	2.00 ± 0.00^b^
Data presented as Mean ± SD. The same letters between the 2 groups show that the 2 groups are not statistically different. P ≤ 0.05 is considered statistically significant. FEO: Fennel essential oil, Mn: Manganese, PCOS: Polycystic ovarian syndrome							

## 4. Discussion

In this study, we examined the effects of FEO and Mn on hormonal and oxidative stress markers and also ovarian morphology, in a PCOS rat model. The experimental design included 7 groups: control (normal saline), sesame oil, FEO, Mn, PCOS, PCOS+FEO, and PCOS+Mn. Examining each group in detail help us to investigate and discuss the reasons behind the results that we observed.

As expected, the control group, with the lowest levels of estrogen, progesterone, and MDA, did not develop ovarian cysts, substantiating its function as a control. The sesame oil group which served as a vehicle for EV, showed a slight rise in hormone levels without the development of cysts. In the non-PCOS group that received FEO, estrogen and progesterone levels rose significantly and the number of ovarian cysts paradoxically increased as well. This indicates the presence of phytoestrogens in FEO that excessively activates estrogen receptors. Such results could discourage the consumption of FEO among normal individuals without prevailing conditions, as FEO might have cyst-inducing potential based on previous studies (21). The Mn group had higher levels of progesterone, likely due to antioxidant properties and potential role in LH stimulation, proposing Mn's involvement in improving ovarian activity. In the PCOS group, elevated levels of estrogen and MDA with high incidence of ovarian cysts, confirmed the successful modeling of the PCOS condition in rats, providing a foundation for evaluating the therapeutic effects of FEO and Mn. The PCOS+FEO group demonstrated a significant decrease in the number of cysts, increased hormone levels, and reduced markers of oxidative stress that highlights FEO's estrogen-modulating and antioxidant properties in PCOS. Nevertheless, the cyst-inducing activity in the non-PCOS rats still remain a cause for concern. The results in the Mn+PCOS group indicated a significant decrease in cysts along with an increase in progesterone levels, but estrogen levels remained constant, probably due to the antioxidant activity of Mn combined with its influence on LH pulses. These findings show that Mn may be employed as a therapeutic strategy for managing PCOS.

Confirmation of PCOS induction was achieved through microscopic examination of ovarian tissue with a notable increase in cyst count. PCOS was induced by a single dose of EV, following established protocols (22). After 60 days, tissue analysis confirmed EV's prolonged estrogenic effects disrupted LH secretion and gonadotropin-releasing hormone regulation (23). The PCOS group exhibited larger and more ovarian cysts in comparison with the control group. Conversely, neither the control nor sesame oil groups exhibited cysts due to the absence of risk factors.

Interestingly, FEO alone increased cyst counts even more than estradiol. FEO, as a phytoestrogen may induce cyst formation in healthy ovarian tissue due to its estrogenic compounds (21), particularly trans-anethole, which has structural similarities with diethylstilbestrol, a synthetic estrogen (18). This suggests a potential risk for normal women without ovarian cysts who use FEO for osteoporosis or menstrual relief. More research need to be conducted to validate this claim. On the other hand, in PCOS-induced rats, FEO significantly reduced cyst counts, which suggests its therapeutic potential for ovarian cysts. Our findings align with previous studies in which FEO reduced cystic follicles (14, 24), improved ovarian histology, and enhanced ovulation rates by influencing folliculogenesis (25). Phytoestrogens in FEO may inhibit receptor access under high endogenous estrogen conditions like PCOS (26), while trans-anethole's strong binding to androgen receptors points to its viability as a therapeutic agent for PCOS (21). Similarly, Mn supplementation in PCOS rats significantly reduced cyst counts. Mn is a trace element contributing to oxidative stress and glucose metabolism in PCOS (1). Animal studies have shown that the supplementation of Mn decreases ovarian cysts and increases corpus luteum formation (15, 27).

PCOS is influenced by genetic and environmental factors that cause hormonal imbalances, particularly estrogen and progesterone (25) which regulate the menstrual cycle and ovulation. In PCOS, adipose tissue and ovaries overproduce estrogen, while low progesterone results from irregular ovulation and reduced corpus luteum. This study found that estrogen and progesterone levels were significantly higher in PCOS rats treated with FEO compared to other groups which is in line with previous research (17). These hormonal changes are associated with phytoestrogens like anethole and antiandrogens such as β-sitosterol and palmitic acid that highlight the fennel's role in managing hormonal imbalances in PCOS (25). In this regard, phytoestrogenic compounds in fennel can attach to estrogen receptors because they structurally resemble estradiol. These compounds mimic or block estrogen effects by binding to estrogen receptors (28) and regulating estradiol synthesis via the hypothalamus-pituitary-gonadal axis (29). Additionally, fennel's hormone-stimulating properties are because of compounds like estragole and apiole, elevate estrogen levels (30). Studies confirm that fennel extract increases estrogen levels and exhibits estrogenic effects in rats (31) because of aromatic compounds like dianethole, photoanethole, fenchine, and p-anisaldehyde (32). Hence, the increase in estrogen levels in the PCOS+FEO group may be related to these findings.

In PCOS, decreased progesterone levels result from anovulation and fewer corpora lutea (33). Elevated progesterone in the PCOS+FEO group may be a potential asset in treating PCOS. Studies show fennel increasing serum progesterone levels in PCOS rats (34), and fennel seed powder (2–4%) in female rat diets elevating progesterone levels, support this assertion (35). This effect may result from phytoestrogens inhibiting 20 α -hydroxysteroid dehydrogenase which may decrease progesterone metabolism (36). These findings explain the rise in estrogen and progesterone in the PCOS+FEO group. Top of FormInterestingly, the FEO group showed the lowest progesterone levels after the control which is inconsistent with our finding indicating that 200 mg/kg FEO over 14 days induces ovarian cysts and decreases progesterone. Mn supplementation in PCOS group also elevated progesterone levels compared to control group. This finding is supported by a study showing that LH levels which are positively correlated with increased progesterone levels (37), were increased in PCOS women that were exposed to Mn (38).

In the final section of the study, MDA levels increased in PCOS groups, consistent with findings linking elevated MDA to lipid peroxidation, tissue disruption, ROS production, and increased androgen levels (39). Notably, the lowest MDA level was observed in the PCOS group following the control group, while the FEO group exhibited the highest MDA level and cyst count suggesting that at 200 mg/kg for 14 days, FEO exacerbates oxidative stress and histological conditions even more than EV. Despite the antioxidant properties of fennel, FEO supplementation in PCOS groups may induce a pro-oxidant effect, possibly due to estrogen imbalance or interaction with EV which might alter metabolism or effects of FEO on tissues, further disrupting hormonal balance and exacerbating oxidative stress. While FEO contains antioxidants like anethole, it shows dose-dependent and duration-based effect. Contradictory findings in other studies may result from differences in dosages, methods, or animal models (40). The effects of FEO on MDA levels and oxidative stress in PCOS need to be explored. Mn, which is recognized for its antioxidant properties, did not significantly alter MDA levels in PCOS groups and likely is due to differences in the methods or doses used. To better understand therapeutic potential and limitations, clinical trials and focusing on long-term impacts of FEO and Mn in PCOS patients are necessary, to confirm these results and determine optimal treatment regimens.

## 5. Conclusion

Based on our results, FEO increased estrogen and progesterone levels in PCOS rats while Mn mainly elevated progesterone and had a protective effect against oxidative stress. Considering that the focus in the treatment of PCOS is generally on restoring hormonal balance rather than simply increasing or decreasing one hormone; this goal was well achieved in this study. In histological evaluation, FEO and Mn both decreased ovarian cysts in PCOS groups. Through these results, it appears that FEO and Mn can be used in the treatment of PCOS by reducing ovarian cysts, improving hormonal markers, and alleviating oxidative stress, although via different mechanisms. The combination of Mn and FEO provides a multifaceted approach of the PCOS treatment. However, the optimal dose, treatment duration, and safety must be determined.

##  Data Availability

Data supporting the findings of this study are available upon reasonable request from the corresponding author.

##  Author Contributions

H. Najafzadehvarzi and SM. Ghoreishi designed the study. H. Najafzadehvarzi, E. Mohebbi Kian, and M. Barancheshmeh equally performed the experiments. E. Mohebbi Kian, M. Barancheshmeh, SM. Ghoreishi, and N. Shokrzadeh contributed to data collection, evaluation, drafting, and statistical analysis. All authors reviewed and approved the final manuscript and take responsibility for the integrity of the data.

M. Barancheshmeh and E. Mohebbi Kian are both co-first author due to their equal contributions. M. Barancheshmeh conducted the animal work, drug treatments, microscopy, and histological assessments, while E. Mohebi Kian performed hormonal and MDA analyses. Their tasks were divided based on availability due to geographical constraints.

##  Conflict of Interest 

The authors declare that there is no conflict of interest.
